# Derationalizing Delusions

**DOI:** 10.1177/2167702620951553

**Published:** 2020-11-20

**Authors:** Vaughan Bell, Nichola Raihani, Sam Wilkinson

**Affiliations:** 1 Research Department of Clinical, Educational and Health Psychology, University College London; 2 Psychological Interventions Clinic for Outpatients with Psychosis, South London and Maudsley NHS Foundation Trust, London, England; 3 Department of Experimental Psychology, University College London; 4 Department of Sociology, Philosophy and Anthropology, Exeter University

**Keywords:** delusion, psychosis, schizophrenia, belief

## Abstract

Because of the traditional conceptualization of delusion as “irrational belief,” cognitive models of delusions largely focus on impairments to domain-general reasoning. Nevertheless, current rationality-impairment models do not account for the fact that (a) equivalently irrational beliefs can be induced through adaptive social cognitive processes, reflecting social integration rather than impairment; (b) delusions are overwhelmingly socially themed; and (c) delusions show a reduced sensitivity to social context both in terms of how they are shaped and how they are communicated. Consequently, we argue that models of delusions need to include alteration to coalitional cognition—processes involved in affiliation, group perception, and the strategic management of relationships. This approach has the advantage of better accounting for both content (social themes) and form (fixity) of delusion. It is also supported by the established role of mesolimbic dopamine in both delusions and social organization and the ongoing reconceptualization of belief as serving a social organizational function.

Delusions are defined as wildly improbable beliefs that are strongly held despite incontrovertible and obvious counterevidence and despite what almost everyone else believes (American Psychiatric Association, 2013). Although the precise definition of delusions has been much debated (Bell et al., 2003; Coltheart et al., 2011; David, 1999), they are considered the archetypal characteristic of “madness” and a central component in the diagnosis of psychotic disorders such as schizophrenia, delusional disorder, and schizoaffective disorder; they are also thought to emerge after a range of neurological disorders and alterations to the central nervous system (Gilleen & David, 2005).

Dominant historical and contemporary approaches have conceptualized delusions as pathological beliefs that are characterized by their irrationality (Bayne, 2017; Berrios, 1991; Bortolotti, 2005; Sakakibara, 2016). Consequently, current cognitive theories have largely focused on describing impairments to general-purpose inferential reasoning as the sole or central part of the explanatory model of delusions (Bell et al., 2006; Broyd et al., 2017; Feeney et al., 2017). Existing cognitive models have variously attempted to explain this through multifactorial impairments to probabilistic reasoning, perception, and affect (Garety & Freeman, 1999); a two-stage impairment to perception and reasoning (Langdon & Coltheart, 2000); aberrations in the use of prediction error in hierarchical inference mechanisms (Corlett et al., 2010); impairments to various aspects of metacognitive representation and control (Bronstein et al., 2019; Frith et al., 2000; Moritz & Woodward, 2006); or a synthesis of several of these approaches (e.g., Broyd et al., 2017; Miyazono & McKay, 2019).

However, we argue here that this characterization—that privileges the explanation of irrationality at the expense of explaining the social form of delusions—has hindered researchers’ understanding. Specifically, these approaches have focused on finding problems with the components of domain-general rationality and have largely disregarded the potential role of socio-cognitive dysfunction. This approach has seemed viable only by failing to consider that healthy and adaptive social processes can form and maintain delusion-like beliefs by ignoring the most striking phenomenological characteristics of delusions—that they are overwhelmingly socially and relationally themed—and by disregarding the fact that delusions show a reduced sensitivity to social context both in terms of how they are shaped and how they are communicated.

In line with recent work on the social organizational function of belief (Boyer et al., 2015; Echterhoff et al., 2009; Gelpi et al., 2019; Jost et al., 2008; Mercier & Sperber, 2011; Williams, 2020), we suggest that causal models of delusions need to include dysfunction to coalitional cognition—that is, social cognitive processes involved in social influence, affiliation, interaction with groups, and the management of relationships. This approach has the advantage of better accounting for aspects of both the form and content of delusional beliefs as well as better reflecting the known function of the mesolimbic dopamine system—which is involved in the formation and maintenance of delusions and the management of social hierarchy, dominance, and cooperation.

## The Missing Irrationality in Delusions

On face value, it might be reasonably assumed that someone who is distressed and disabled by the belief that they are dead (Sahoo & Josephs, 2017), or that a camera has been implanted in their tooth and is taking pictures of their mind (Tanenberg-Karant et al., 1995), or that they are being poisoned by the CIA because they are Jesus (Mitchell & Vierkant, 1991) has a problem with rationality.

Although some approaches have suggested that delusions are solely explained by a problem with perception (Maher, 1999) or language (Hinzen et al., 2016), the majority of cognitive accounts draw on the long-established historical idea that delusions reflect an impairment to rationality (Berrios, 1991), which now forms one of the central tenets of explanation for delusional beliefs (see reviews in Bortolotti, 2010; Gold & Hohwy, 2000; Leeser & O’Donohue, 1999; Radden, 1985). This approach suggests that the presence of marked epistemic irrationality (i.e., beliefs that wildly deviate from the conclusions generated by normative principles of reasoning given available information), usually in addition to marked procedural rationality (i.e., inconsistency with clearly relevant and available preexisting beliefs and mental states given the application of reliable forms of inference; Bermúdez, 2001), implies problems with the cognitive processes underlying the capacity to reason pragmatically (Gerrans, 2001). Note that the standard of comparison is normative because cognitive biases are considered to be an integral part of healthy, adaptive cognitive functioning (Bayne, 2017; Johnson et al., 2013). Consequently, this has motivated researchers to look for differences in performance on reasoning tasks that are assumed to reflect underlying impairments in domain-general rationality (Garety, 1991).

Initial assumptions that delusional beliefs reflect an impairment in syllogistic (i.e., deductive) reasoning (Von Domarus, 1944) were discredited because numerous studies failed to find any generalized difficulty in syllogistic reasoning associated with delusional beliefs when compared with IQ-matched control participants (Mujica-Parodi et al., 2000). Indeed, this is clearly apparent during conversation with someone with delusions. All things being equal, they can generate normative conclusions about matters not relating to their delusional beliefs and come to seemingly atypical conclusions only when reasoning relates to the specific focus of the delusions. Therefore, most people with delusions appear not to have a generalized problem with rationality but a circumscribed irrationality by which conclusions are irrational solely in relation to the delusional content (Gold & Hohwy, 2000).

It could be argued that one potential source of impairment to rationality might arise from general cognitive difficulties that are common in patients diagnosed with psychotic disorders (Bora et al., 2010). However, there is just a weak correlation between general cognitive performance and the presence of positive symptoms in patients—of which delusions are a central feature (Ventura et al., 2013). There is also a significant effect of referral bias in that community epidemiological studies show much smaller cognitive differences between people with and without psychosis than clinical studies (Mollon et al., 2016). Furthermore, cognitive impairment is neither necessary nor sufficient for the production of delusions because the vast majority of patients with even quite severe cognitive difficulties, for example after traumatic brain injury, do not develop delusions as a consequence and indeed do not show rates that markedly differ from the general population (Ponsford et al., 2018). Clearly, some delusions arise after brain injury, dementia, or alterations to the nervous system, for example, but there are no reliable instances of cognitive impairment independent of etiology that reliably raise the risk of developing delusions.

One potential explanation for this is that delusions involve a more subtle type of cognitive alteration that is detectable but must interact with other difficulties, such as perceptual dysfunction, emotional dysregulation, traumatic life events, and maladaptive coping, to form and maintain delusional belief (e.g. Garety et al., 2001; Langdon & Coltheart, 2000).

Consequently, various cognitive biases have been proposed as important in the development or maintenance of delusions. The most studied has been the “jumping to conclusions” probabilistic reasoning bias, most commonly tested using the beads task (Garety, 1991; Huq, Garety, & Hemsley, 1988). One recent large-scale study of people with first-episode psychosis reported that the jumping to conclusions bias was reliably associated with psychosis (Tripoli et al., 2020), although this was entirely accounted for by general cognitive difficulties and not the presence of psychotic symptoms per se. Recent meta-analyses (Dudley et al., 2016; So et al., 2016) reported that patients with current delusions drew fewer beads than patients without delusion, but this effect when quantified was 0.6 fewer beads, and 40% to 50% of patients showed no evidence of the bias whatsoever. Other cognitive biases associated with delusions show a similar pattern. A recent meta-analysis (McLean et al., 2017) of a range of cognitive biases in delusions, including jumping to conclusions bias, bias against disconfirmatory evidence, bias against confirmatory evidence, and liberal acceptance bias, showed small to moderate effect sizes when comparing patients with and without schizophrenia but did not include control participants for general cognitive function.

We note here that research motivated by the search for evidence of problems in domain-general rationality has had some success, although the effects are relatively small in light of the striking deviations from normative conclusions about reality represented by the presence of delusions, suggesting an ongoing “validity gap” in the rationality-impairment approach.

## Delusion-Like Beliefs Arise From Unimpaired, Adaptive Social Processes

In rationality-impairment approaches, the irrationality of beliefs is typically evaluated in terms of normative conclusions about the natural world (i.e., in terms of epistemic irrationality) and not in terms of the role the belief plays in the social environment. For example, it may be “irrational,” given the natural world, for people to believe their thoughts control the weather, but it may be socially beneficial given that, if this belief is strongly held by their social group, rejecting it may lead to hostility and ostracism with potentially far greater negative consequences (Edis & Boudry, 2019; Rauwolf et al., 2015), which suggests the belief may be “functionally rational” (Bayne, 2017).

Indeed, there are many examples of how strongly held, affectively loaded, epistemically irrational beliefs can be reliably formed through social influence—demonstrating that adaptive social processes, rather than individual impairments in rationality, are sufficient to form delusion-like beliefs in cognitively intact people. We document several examples below.

The sudden spread of intense delusion-like ideas through the population are well documented and frequently occur over time and across cultures and have variously been called “mass delusion” and “social delusion” and have been studied alongside “mass hysteria” (Bartholomew, 2001). Episodes of socially transmitted delusion-like belief outbreaks have included beliefs in a “phantom anaesthetist” who was believed to be prowling the community and “gassing” members of the public (Bartholomew & Victor, 2004), a “windshield pitting epidemic” attributed to nonexistent “H-bomb tests” (Medalia & Larsen, 1958), frequent waves of beliefs about malicious penis-stealing episodes (Dan et al., 2017), the kidnapping of children to use their decapitated heads in the foundations of new buildings (Barnes, 1993), and an invasion of foreign airships before capable technology was available (Holman, 2016), to name but a few. Note that these are distinct from conspiracy theories in that rather than being “explanations for important events that involve secret plots by powerful and malevolent groups” (Douglas et al., 2017, p. 58), they typically involve new and idiosyncratic phenomena that reflect a direct and personal risk to the individual believer. These episodes of mass social delusion typically include thousands of people and motivate urgent behavior designed to protect the individual and community, but they resolve quickly, usually within weeks, as the community interest wanes (Bartholomew, 2001).

Intense delusion-like beliefs that greatly deviate from both consensual reality and orthodoxy have been reported in members of small, isolated religious groups. These groups can be cults, which can involve coercive control (Rodríguez-Carballeira et al., 2015), but also more benign groups in which membership is through enthusiastic and voluntary participation (Dawson, 1996). However, common to both is intense within-group contact and the reduction of extragroup social ties (Coates, 2012). Examples of firmly and intensely held, improbable, behavior-shaping beliefs in members of noncoercive isolated religious groups include that mass suicide (eventually carried out) would allow group members to meet an alien space ship accompanying the Hale-Bopp comet, which would cause them to ascend to “The Evolutionary Level Above Human” (Robinson, 1997); that a forthcoming nuclear war would wipe out a third of the world’s population and the group members would be subsequently gifted miraculous powers of healing to save humanity (Melton, 1985); that God would appear on a specific cable TV channel to announce his presence to the world (Prather, 1999); and that group members were under attack from electromagnetic wave warfare designed to disrupt their spiritual work, which could be deflected by wrapping themselves in white cloth (Murguia, 2011).

The transfer of delusional beliefs from one person to others has been variously classified as *folie à deux, induced delusional disorder*, and *shared psychotic disorder* (Shimizu et al., 2007). This can take several forms, but the type most relevant here is one in which a secondary person who is not otherwise experiencing psychosis takes on the delusional beliefs of the first person who has psychosis. It is now recognized that many secondary subjects of induced delusional disorder may have risk factors for psychotic disorder—genetic risk factors because relatedness is essential (Arnone et al., 2006). However, there are also clear cases of induced delusional disorder in secondaries who do not share relevant risk factors, including taking on the delusion of a primary with dementia that the house was being continually burgled by neighbors who would replace the items shortly after stealing them (Brooks, 1987); a husband, two sons, sister-in-law, and nephew taking on a delusion that the neighbors were using technology to persecute them through the ceiling (Dippel et al., 1991); and parents taking on the delusion resulting from amphetamine psychosis of the son that he was being persecuted, his walls were bugged, and he could detect which bricks had listening devices in them because of “their different look” (Hill et al., 2001). Relevant nonbiological risk factors for induced delusions include social isolation (José & Mary, 1995), the length and closeness of the relationship (Arnone et al., 2006), and enmeshed and dependent relationship style (Mentjox et al., 1993).

Experimentally, delusional beliefs have been modeled, albeit transiently, in the lab using hypnosis that involves a combination of suggestion and active engagement of highly suggestible individuals (Cox & Barnier, 2010). More prosaically, although orthodox religious beliefs are complex and include dogma relating to social organization as well as reality, they have a poor historical track record of reflecting accurate models of the natural world but have been successfully transmitted to large numbers of people (Edgell, 2012).

These examples demonstrate that healthy and adaptive social processes have a powerful role in belief formation—to the point that social influence can form and maintain beliefs that are as epistemically irrational, affectively loaded, and strongly held as delusional beliefs. However, that these beliefs wane or modify as the social context changes and reflect successful social integration in context of the community or microcommunity in which they originate rather than social disability suggests they reflect adaptive rather than impaired functioning. Therefore, these examples also demonstrate that privileging irrationality is unlikely to be an effective explanatory strategy for models of delusional belief because it will not successfully account for their maladaptive nature.

## The Majority of Delusions Have Social Themes

Delusions have a relatively select number of themes, with social themes being overwhelmingly the most common presentation. Despite this clear social phenomenology, the majority of cognitive models of delusions do not consider it a focus for, or a constraint on, explanation.

Common delusional themes include persecution, reference, guilt or sin, grandiosity, erotomania, jealousy, somatic changes, religion, mind reading, external control, thought broadcast, insertion, and withdrawal (Gutiérrez-Lobos et al., 2001; Paolini et al., 2016; Peralta & Cuesta, 2016). Delusions of persecution, reference, jealousy, and erotomania are social because, by definition, they implicate social actors. Other delusion types are not by definition socially themed but commonly present as social. Grandiose delusions often involve beliefs about having an elevated social position, important social role, or link to important people (Suhail & Cochrane, 2002). Delusions of guilt or sin typically involve concerns about contravening the expectations of others, including spiritual beings (Stompe et al., 1999). Religious delusions often involve a spiritual identity determining relationships with others or a closeness or relationship with spiritual figures (Rieben et al., 2013). Delusions of external control, thought insertion, broadcast, and withdrawal typically involve concerns about the social permeability of the private mental experience (Schimansky et al., 2012) and being controlled by others (Hirjak et al., 2013). Delusions as a whole also commonly include beliefs about illusory social agents that seem present across themes (Bell et al., 2017).

Epidemiologically, persecutory delusions are by far the most common presentation in nonaffective psychosis (de Portugal et al., 2013; Ellersgaard et al., 2014), psychosis in Parkinson’s disease (Warren et al., 2018), and drug-induced psychoses (Voce et al., 2019). In the affective psychoses, grandiose delusions or delusions of guilt or shame may be more common depending on mood state (Picardi et al., 2018). Indeed, one of the notable attributes of delusions as a whole is that the majority involve key themes likely to reflect adaptive social processes shaped by evolution—for example, danger from others, social position, social stigma, and social affiliation (Bortolotti, 2015; McKay & Dennett, 2009).

Despite this, the majority of cognitive models do not attempt to address why delusions are typically socially themed rather than not socially themed. Several cognitive models exclude specific alterations to social cognition and do not address the social theming of delusions (Bronstein et al., 2019; Broyd et al., 2017; Feeney et al., 2017; Frith et al., 2000); several suggest that content, including social content, is determined by anomalous experience of which only specific examples are given (e.g., reduced autonomic responding to familiar people in Capgras delusion), which cannot account for the prevalence of social themes in delusions more generally (Langdon & Coltheart, 2000; McKay, 2012); whereas others restrict themselves to a specific social theme, typically persecutory delusions (Bentall et al., 2001; Freeman, 2016), and so cannot explain why delusions per se have social rather than nonsocial themes.

It is therefore clear that current cognitive models of delusional belief do not attempt to, or cannot, account for the most common thematic content of delusional beliefs.

## Delusions as Dysfunction in Coalitional Cognition

Given (a) the modest findings from studies searching for domain-general impairments to rationality, (b) the demonstrable role of adaptive social processes in the formation of delusion-like irrational beliefs in healthy individuals, (c) the social themes of the vast majority of delusions, and (d) the reduced sensitivity of delusions to social context, we suggest that social cognitive processes are an important candidate for inclusion in any adequate cognitive model of delusions. This raises the question of how to conceptualize the role of social cognition in such models.

One potential objection to the inclusion of social cognitive mechanisms in models of delusions is that traditional measures of social cognition barely predict the presence of reality distortion symptoms of psychosis, tending to predict negative symptoms more strongly (Ventura et al., 2013). However, we agree with previous authors that the most widely used measures of social cognition do not capture the full range of relevant cognitive processes needed to explain disturbed social experience in psychopathology (Gallagher & Varga, 2015; Schilbach, 2016; Yager & Ehmann, 2006). Indeed, traditional measures tend to require individual testing of participants while exposing them to short instances of static social information that largely quantify individualized mechanisms of social information processing (M. F. Green et al., 2015) rather than the management of dynamic, evolving, multiagent social situations (Bell et al., 2017; Schilbach, 2016).

Instead, we suggest that processes involved in social influence, affiliation, and strategic social behavior are likely to be key in understanding delusions. Cognitive processes focused on garnering support from other individuals, organizing and maintaining alliances, and perceiving social status in groups have been described as “coalitional psychology”—something that is highly likely to be part of evolved cognitive system adapted to life in complex social groups (Boyer et al., 2015; Tooby & Cosmides, 2010). Evidence for the evolutionary basis of this system stems from the fact that life in complex social groups favors the evolution of specialized and sophisticated socio-cognitive abilities (Dunbar & Shultz, 2017) and that the ability to form and maintain coalitional alliances is present in multiple species and throughout hominid evolutionary history (Kappeler & van Schaik, 2002).

We cite coalitional cognition as important in delusions for several reasons. First, beliefs are now increasingly recognized as strongly or primarily social in function, which implies that delusions need to be understood in terms of the same social mechanisms. Recent work in social cognition has suggested that conscious access to metacognitive judgments such as beliefs exists largely, if not primarily, to facilitate social interaction and group coordination (Tomasello, 2009). Frith (2012b) described how complex social coordination requires explicit communication of metacognitive judgments that allows for group refinement of knowledge and joint action. Likewise, Mercier and Sperber (2011) argued that the primary function of reasoning is not for the refinement of personal knowledge, given the extensive evidence for how ineffective it is for this purpose, but for argumentation, social communication, and persuasion. Here, beliefs, reasons, and knowledge primarily are generated to communicate what are usually intuitive inferences for group refinement and social coordination. Cushman (2019) argued that the post hoc generation of reasons may not be efficient in itself for solving problems, but it allows people to apply the same reflective process to themselves and to others, again facilitating social coordination for problem-solving. Williams (2020) suggested that understanding the social function of belief better accounts for both its function and phenomenology but also better explains phenomena such as confabulation, positive illusions, and identity-protective cognition.

Second, the social themes of delusions are also primarily coalitional in nature and involve concerns about persecution by others, social status, social affiliation, or social transgressions (e.g., McKay & Dennett, 2009; Raihani & Bell, 2019). In addition, delusions frequently involve belief in the existence and actions of illusory social agents that impinge on the life of the believer (Bell et al., 2017). Indeed, we note that delusions are frequently delusional precisely because they cite the actions of coalitions that do not exist or cannot perform the actions believed of them. Moreover, observational studies have reported that delusions typically involve coalitional characteristics that go often beyond the simple theme of the delusion itself. Individuals with persecutory delusions rank their perceived persecutors as having higher social rank than them (C. Green et al., 2006; Paget & Ellett, 2014) and frequently involve a perception of being targeted by a specific group (Raihani & Bell, 2019). Erotomanic delusions frequently involve the belief that a person of higher social status is in love with the believer (Brüne, 2001), whereas delusions of jealousy typically relate to perceived infidelity and the resultant humiliation (Seeman, 2016). Grandiose delusions involve a belief that the individual has risen in social status and has a special mission or message for the world (Knowles et al., 2011). Experimental studies of psychosis and the psychosis spectrum that have examined coalitional perception and behavior have reported alterations in the social perception of groups as paranoia increases (Greenburgh et al., 2019), an impact of in-group/out-group status on attribution of harmful intent in paranoia (Saalfeld et al., 2018), alterations to the social representation of others (Raihani & Bell, 2017), and a range of alterations to cooperative behavior and emotional reactions to differences in cooperative behavior in others accompanying delusions (Ellett et al., 2013; Fett et al., 2012, 2016; Gromann et al., 2013; Savulich et al., 2018).

Third, by definition, delusions are both irrational and show reduced sensitivity to social context. This latter characteristic suggests they may involve a dysfunction in the ability to have beliefs moderated and refined through social interaction and reflect problems with the strategic presentation of beliefs given social context. The criterion that delusions are not beliefs “ordinarily accepted by other members of the person’s culture or subculture” has been maligned as a pragmatic decision to avoid the uncomfortable situation of diagnosing religious or spiritual beliefs as delusional given that many such beliefs would otherwise fulfill the irrationality criteria (Pierre, 2001; Ross & McKay, 2017). But although religious beliefs may be epistemically irrational when judged against how well they explain the natural world, they may still be highly socially beneficial. In contrast, delusions are typically epistemically irrational beliefs that, in addition, are socially maladaptive, indicated by the fact they form outside of a shared community of belief and are resistant to social context, which leads to high levels of social disability and ostracism. Social context effects are not only about the influence of the social milieu on the content and conviction in the belief but also include being able to strategically communicate the belief given the social environment (Fransen et al., 2015). For example, one potential solution to having a belief that leads to social sanction is to keep it to oneself, deny it, or reveal it only to people who are unlikely to sanction the believer (Edis & Boudry, 2019). Although not well studied, this is an ability people with delusions seem to progressively lose as delusions becomes more intense, as suggested by greater difficulties in using social context for pragmatic communication with increasing severity (Colle et al., 2013).

Finally, a coalitional approach to delusions is consistent with the known role of the mesolimbic dopamine system, which has been strongly implicated in both delusions (Howes & Kapur, 2009) and coalitional cognition (O’Connell & Hofmann, 2011; Salamone & Correa, 2012; Trainor, 2011). Increased dopamine turnover in the mesolimbic dopamine system is present in in patients with delusions (Winton-Brown et al., 2014) whereas increasing dopamine turnover by the use of stimulant drugs causes delusions after chronic use (Voce et al., 2019). These same drugs also increase social motivation and sensitivity to emotional expression in nonpsychotic individuals (Wardle & de Wit, 2012; Wardle et al., 2012). Conversely, antagonism of D_2_ dopamine receptors in the mesolimbic pathway by antipsychotic medication reduces the intensity of delusional beliefs (Kaar et al., 2019), reduces social engagement, and induces indifference (Gerlach & Larsen, 1999; Moritz et al., 2013). Furthermore, a wealth of evidence from human and animal studies suggests that the mesolimbic dopamine system mediates various aspects of coalitional cognition, including perception and response to social hierarchy, social defeat, and social dominance (Báez-Mendoza & Schultz, 2013; Ghosal et al., 2019; Krach et al., 2010; O’Connell & Hofmann, 2011). Conversely, there is now increasing evidence that the risk of psychosis is raised by coalitional stress given that social disadvantage and poor social integration (Jongsma et al., 2020) lead to long-term changes in the mesolimbic dopamine pathway (Howes & Murray, 2014; Selten et al., 2013), which suggests a causal pathway that includes coalitional components across levels.

Thus, converging evidence from multiple sources, including observational and experimental studies of delusion, models of normal belief, the social responsiveness of delusional beliefs, and the functions of mesolimbic dopamine, suggests that coalitional cognition is likely an important but currently overlooked component of explanatory models of delusional belief.

## Hypotheses Regarding the Role of Coalitional Cognition in Delusion Formation

In this article, we hypothesize two roles for coalitional cognition in belief formation: (a) underpinning the social processes of belief formation and (b) contributing to the coalitional content of belief. Consequently, we hypothesize that dysfunction to coalitional cognition contributes to two aspects of delusions: first, by affecting the form of delusions—for example, their resistance to social influence—and second, by affecting the content of delusions—resulting in their frequent social themes.

Note that we are hypothesizing that dysfunction to coalitional cognition may play a role in delusions regardless of their theme, not solely socially themed ones, because social belief formation processes apply widely and not solely to socially themed beliefs. However, because coalitional cognition impairment would typically also lead to a misperception of the social environment, socially themed delusions would be the most common type—in line with the results from epidemiological studies.

Consequently, we describe some specific hypotheses regarding dysfunction to coalitional processes that we suggest are candidates for inclusion in delusion-formation mechanisms.

As described above, one common feature of delusions is a reduced ability to strategically communicate (or strategically hide) the belief in social situations. Collective decision-making is common in social species and involves the ability to judge other group members’ information, integrate social markers of reliability and influence (e.g., status), and communicate one’s own decision and confidence given the social context (Bang & Frith, 2017). For humans, one important consideration may be the strategic balancing of personal and group goals, particularly when they are in conflict (Bazazi et al., 2019). We hypothesize that people with delusions will show selective difficulties with the strategic communication of beliefs during group decision-making when the focus relates to delusion content even when it is in their interest to do so. Recently developed paradigms to test these processes seem well suited to this hypothesis, and we suggest that people with psychosis will show delusion-related difficulties communicating beliefs and belief conviction for group decision-making (Bang et al., 2017) and in strategic communication in which differences between felt confidence and communicated confidence are key to task completion (Bang et al., 2020).

With regard to delusion content, cognitive models of persecutory delusions frequently conceptualize the core pathology as overactive social threat perception with no strong distinction made between the processes involved in the perception of individuals and the perception of groups (e.g., Diaconescu et al., 2019; Freeman, 2016; Freeman & Garety, 2014). However, persecutory delusions frequently include not only a belief about increased threat of harm but also a belief in a conspiracy behind the intent to harm, often involving the selective identification of a (seemingly arbitrary) group of persecutors (Raihani & Bell, 2019). As noted above, these sorts of social group misperceptions appear widely in delusions and appear in several delusion subtypes. Cognitively, the mechanisms underlying group perception seem to be distinct from individually focused social cognitive process such as theory of mind (Dunham, 2018), are present across species (Bissonnette et al., 2015), and emerge early in human infancy (Rhodes & Baron, 2019). Consequently, we hypothesize that people with delusions that involve the misperception of social alliances will show difficulties with the accurate perception of group boundaries and the judgment of joint intent, particularly as the task becomes more related to the topic of their delusional concern.

That delusions commonly involve a belief of having a radically altered social status (e.g., C. Green et al., 2006; Isham et al., 2019) and that, independently, social exclusion and social distance are risk factors for psychosis (Jongsma et al., 2020) suggest that cognitive mechanisms involved in the perception and processing of social status may be an important site of pathology. Processes involved in perceiving and managing social status are common across a range of social species, including humans; have an evolutionarily important social function (Chiao, 2010); and do not seem to be explainable solely in terms of mentalization (Rushworth et al., 2013). Social-rank perception has already been identified as important in mediating beliefs about and distress resulting from hallucinated voices (Larøi et al., 2019), and we hypothesize that perceived social-rank differences will have an effect on promoting delusional ideation in the relevant direction (e.g. Saalfeld et al., 2018) and that people with delusions involving marked changes in perception of social status will perform more poorly on tasks that measure of accurate perception of social status within a group.

## Conclusions and Future Directions

We have argued that dysfunction to coalitional cognition is likely to be an important component in any adequate cognitive model of delusions. This approach has several benefits: (a) It suggests how a model that includes dysfunction to social processes that moderate belief formation could better explain aspects of the form (fixity and reduced sensitivity to social context) and content (social themes) of delusions, (b) it distinguishes delusional from nondelusional epistemically irrational beliefs, and (c) it is supported by the role of the mesolimbic dopamine system in both delusions and coalitional cognition.

However, we note several outstanding issues. The first is the relationship between dysfunction to coalitional cognition and dysfunction to other cognitive systems. We suggest that dysfunction to coalitional cognition may be sufficient, in itself, to account for some delusions. Given that well-functioning and adaptive social processes can form delusion-like beliefs, dysfunction to this system may lead to misconstrual of the social environment causing delusions to form without the need for impairment to other cognitive systems. The exact content of the belief (“Madonna is in love with me”) may well be a post hoc rationalization of a less specific social inference that, for example, a high-status person has a romantic connection to a person, with the same dysfunction making it difficult to moderate or socially test the specific belief after it has formed.

Alternatively, there may be an interaction between an impairment in coalitional cognition and impairment to other cognitive systems. Perhaps most convincing in this regard are delusions of alien control that are frequently socially themed but evidence suggests may also depend on dysfunction to mechanisms of action monitoring and execution (Blakemore, Wolpert, & Frith, 2002; Frith, 2012a). Likewise, perceptual dysfunction and hallucination may provide additional content for the delusional belief. However, it is worth noting here that hallucinated voices, the most common hallucinations that accompany delusions, have clear social content in the majority of cases (Bell, 2013; Bell et al., 2017; Woods et al., 2015). Likewise, visual hallucinations, also common in psychosis, are most commonly social in content (Collerton et al., 2016; van Ommen et al., 2019). We do not assume all perceptual disturbances related to delusions will have clear social content, but we note the strong thematic similarity, suggesting altered coalitional cognition may also affect perceptual experience.

Furthermore, although most delusions have a clear social theme, a minority do not. Delusional parasitosis, the delusional belief you are infested with parasites, can include delusions about other people as agents of infection, but most cases in the literature simply report a belief about infestation accompanied with perceptual disturbance in the form of formication, a sensation of crawling under the skin (Campbell et al., 2019). Avoidance of infection and contamination have been cited as basic mechanisms underlying social stigma and disgust, and this has been cited as a factor in delusional parasitosis (Kupfer & Fessler, 2018), but it is certainly the case that delusional parasitosis does not seem to be a prima facie example of a socially themed delusion. Likewise, somatic and apocalyptic delusions are not by definition social, although the extent to which these are best understood as reflecting coalitional themes requires further investigation.

In terms of scope, it is clear that future research needs to orient toward the coalitional features of delusions and should deploy methods that better capture coalitional processes effectively. Note that most cognitive models of delusions have either been “generalist,” aiming to explain the formation and maintenance of all delusions without particular reference to their theme, or have restricted themselves to specific themes (e.g., paranoid, external control) in a way that precludes the need to explain why delusions in general have a social theme. Research that takes a comparative approach to delusional themes is still lacking, and we note that some important and common delusional themes (e.g., grandiose delusions) have received remarkably little research attention (Knowles et al., 2011).

Furthermore, additional focus needs to be paid to the coalitional phenomenology of delusions. For example, we have noted that explanations of paranoid delusions mostly characterize them as “delusional fears about harm to self” despite the fact that they frequently include concerns about being persecuted by specific groups (Raihani & Bell, 2019). Delusions, and indeed hallucinated voices, seem to frequently involve illusory social agents, but this social aspect of the experience has been, until recently, markedly underresearched (Bell et al., 2017).

Likewise, many current paradigms in social cognition research are poorly able to capture coalitional processes such as evaluating relationships and alliances or tracking agent identity and status. Social cognition research in psychosis and schizophrenia has broadly coalesced on studying processes related to perception of individual others in ways that largely stem from methods that test individuals by exposing them to social information or involving them in simulated social situations (M. F. Green et al., 2015). Paradigms that involve experimental studies of interaction between individuals (e.g., “second person neuroscience”; Schilbach, 2016; Schilbach et al., 2013) or that examine interaction with groups (e.g., Greenburgh et al., 2019) are likely to be key in capturing the mechanisms of social influence and alliance.

In summary, we argue for a move away from attempts to explain delusions rooted primarily in terms of individual irrationality and instead toward models that include dysfunction to coalitional cognition as an essential component, which we argue are urgently needed to better capture the known form, content, and mechanisms of delusional beliefs.

## Transparency

*Action Editor:* Michael F. Pogue-Geile

*Editor:* Scott O. Lilienfeld

*Author Contributions*

V. Bell drafted the manuscript, and N. Raihani and S. Wilkinson provided critical revisions. All of the authors approved the final manuscript for submission.
